# Exposure to single-walled carbon nanotubes differentially affect in vitro germination, biochemical and antioxidant properties of *Thymus daenensis* celak. seedlings

**DOI:** 10.1186/s12870-023-04599-9

**Published:** 2023-11-20

**Authors:** Saba Samadi, Mohammad Jamal Saharkhiz, Majid Azizi, Leila Samiei, Mansour Ghorbanpour

**Affiliations:** 1 Department of Horticultural Science, Faculty of Agriculture, Shiraz University, Shiraz, Iran; 2 Medicinal Plants Processing Research Center, Shiraz University of Medical Sciences, Shiraz, Iran; 3 Department of Horticulture, College of Agriculture, Ferdowsi University of Mashhad, Mashhad, Iran; 4 Department of Ornamental Plants, Research Center for Plant Sciences, Ferdowsi University of Mashhad, Mashhad, Iran; 5 Department of Medicinal Plants, Faculty of Agriculture and Natural Resources, Arak University, Arak, 38156-8-8349 Iran; 6 Institute of Nanoscience and Nanotechnology, Arak University, Arak, 38156-8-8349 Iran

**Keywords:** SWCNTs, TEM, Thyme, Germination, DPPH, Toxicity

## Abstract

Carbon nanomaterials such as single-walled carbon nanotubes (SWCNTs) offer a new possibility for phyto-nanotechnology and biotechnology to improve the quality and quantity of secondary metabolites in vitro*.* The current study aimed to determine the SWCNTs effects on Thyme *(Thymus daenensis* celak.) seed germination. The seedlings were further assessed in terms of morphological and phytochemical properties. Sterile seeds were cultured in vitro and treated with various concentrations of SWCNTs. Biochemical analyses were designed on seedling sample extracts for measuring antioxidant activities (AA), total flavonoids (TFC) and phenolic contents, and the main enzymes involved in oxidative reactions under experimental treatments. The results indicated that an increase in SWCNTs concentration can enhance the total percentage of seed germination. The improvement was observed in samples that received SWCNTs levels of up to 125 µg ml^−1^, even though seedling height and biomass accumulation decreased. Seedling growth parameters in the control samples were higher than those of grown in SWCNT-fortified media. This may have happened because of more oxidative damage as well as a rise in POD and PPO activities in tissues. Additionally, secondary metabolites and relevant enzyme activities showed that maximum amounts of TPC, TFC, AA and the highest PAL enzyme activity were detected in samples exposed to 62.5 µg ml^−1^ SWCNTs. Our findings reveal that SWCNTs in a concentration-dependent manner has different effects on *T. daenensis* morphological and phytochemical properties. Microscopic images analysis revealed that SWCNTs pierce cell walls, enter the plant cells and agglomerate in the cellular cytoplasm and cell walls. The findings provide insights into the regulatory mechanisms of SWCNTs on *T. daenensis* growth, germination and secondary metabolites production.

## Introduction

In recent years, carbon nano materials have drawn substantial attention because of their great potential for alleviating various problems in food science and plant production [1, 2]. Carbon nanotubes (CNTs) are known to have desirable and undesirable effects on plants [3]. For instance, the previous studies on multi-walled-CNTs (MWCNTs) that MWCNTs can increase water uptake, growth parameters and phytochemicals/active ingredients content in exposed seedlings [4, 5]. Moreover, McGehee et al. [6] claimed that under dark conditions CNTs exposure can enhance *Catharanthus roseus* callus proliferation. The aforesaid study also revealed that CNTs improved alkaloids accumulation. As claimed by Khodakovskaya et al. [7], using CNTs on tomato (*Lycopersicon esculentum*) plants can develop growth parameters and activate over-expressed stress-responsive genes. Khodakovskaya et al. [8] described that using CNTs can improve the fruit numbers on tomato plants. In addition, it is well-known that the presence of CNTs in vitro can change tomato fruits metabolome [9] and transcriptome [7].

*Thymus daenensis* Celak (Thyme, Avishan-e-denaii in Persian) is one of the Iranian endemic medicinal plants, commonly used in traditional medication. It has features that make it anti-inflammatory, tonic, carminative and digestive, due to its valuable compounds including phenolic, flavonoids and essential oils [10]. Pharmaceutical and industrial demands for *T. daenensis* are continuously increasing, and there is mounting pressure on the ecological niche of this species. However, collecting plants from their natural habitats is not scientifically reliable because of variations in phenotypes and genotypes, which mean differences in their secondary metabolites [11]. Meanwhile, culturing these plants in vitro as a climate-free and season-free technique can significantly optimize the uniformity of natural components that are to be derived from the plants. Also, biotic and abiotic elicitors can facilitate the biosynthesis of natural compounds among tissue-cultured plants [12, 13].

One of the most efficient strategies for incrementally enriching the yields of bioactive secondary metabolites such as flavonoids, phenolics acids, carotenoids and alkaloids in plant cells and culture systems has been demonstrated to be elicitation through external chemicals exposure [14, 15]. It has been noted that elicitors can stimulate plant cells by signaling molecules, thereby acting as a functional strategy for biosynthesis and accumulation of secondary metabolites in plants in vitro [16, 17]. Numerous types of nanomaterials have recently been emerged as strong elicitors in medicinal plants, and have been the focus of extensive research by scientists and institutions worldwide [18, 19]. This is mainly due to their unique physio-chemical properties such as small size, which allows them to easily penetrate cells via different pathways, and distribute throughout the tissues, interacting with cellular components and triggering defensive responses in plants [20]. CNTs have been reported to improve germination parameters, vigor index, growth traits, biomass accumulation, cell cycle regulation, agronomical parameters and yield performance [21]. For instance, in soybean the utilization of SWCNTs has led to improvements in the germination index and root and shoot lengths [22]. SWCNTs possess the potential to activate secondary metabolism in *Hyoscyamus niger* due to their ability to easily enter plant cells via seed priming, which help plant overcome and survive adverse conditions [23]. However, several other studies have highlighted the possible toxicity of CNTs in plant cells [24, 25]. It has been acknowledged that the same anomaterials that can stimulate plant growth at lower concentrations may cause toxicity at higher doses [26]. CNTs are believed to penetrate cellular membrane and cause oxidative stress to the cell by promoting the formation of reactive oxygen species (ROS) such as hydrogen peroxide, superoxide, hydroxyl radical, and singlet oxygen, etc. [27]. This process is triggered by the up-regulation of specific enzymes such as NADPH oxidases, peroxidases (POD) and phenylalanine ammonia-lyase (PAL), thereby affecting the phenylpropanoid pathway and the production of secondary metabolites in plants [28]. Previous accounts of research have revealed that CNTs could serve as messengers, change secondary metabolites production and modify physiological responses in many plants [6]. However, information on this specific field is scanty. Also, little is known about the effects of CNTs on endemic plant species. Therefore, the capability of CNTs in causing secondary metabolites accumulation in endemic species of medicinal plants needs to be further studied. An attempt was made in the current research to understand in what way SWCNTs concentrations can change *T. daenensis* seed germination, seedling biomass, along with physiological and phytochemical responses. Specifically, measurements are aimed at phenolics acids and flavonoids content and antioxidant activities. Furthermore, the present study deals with evaluations of cellular modifications in seedling roots and further discusses the fate of SWCNTs using Transmission electron microscopy (TEM) analysis.

## Materials and methods

### Chemicals

SWCNTs were obtained from Nanosany (Nanomaterial’s Pioneers Company, Iran). Also, 2, 3, 5 Theriphenyl Tetrazolium Chloride (TTC), 2, 2´- diphenyl-1- picrylhydrazyl (DPPH.) free radicals, Gallic acid (GAE), Quercetin, Catechol, L- phenylalanine were bought from Sigma Aldrich (Germany).

### Plant materials

*T. daenensis* were collected from their natural niches in Iran. After collection, thyme mature seeds were separated for the next analysis. The identification and authentication of thyme as a species was done by Prof. Dr. M.J. Saharkhiz according to botanical standards [29] and vouchered in Khwarizmi University Herbarium (A66).

### Pristine SWCNTs characterization

**Fig. 1:**
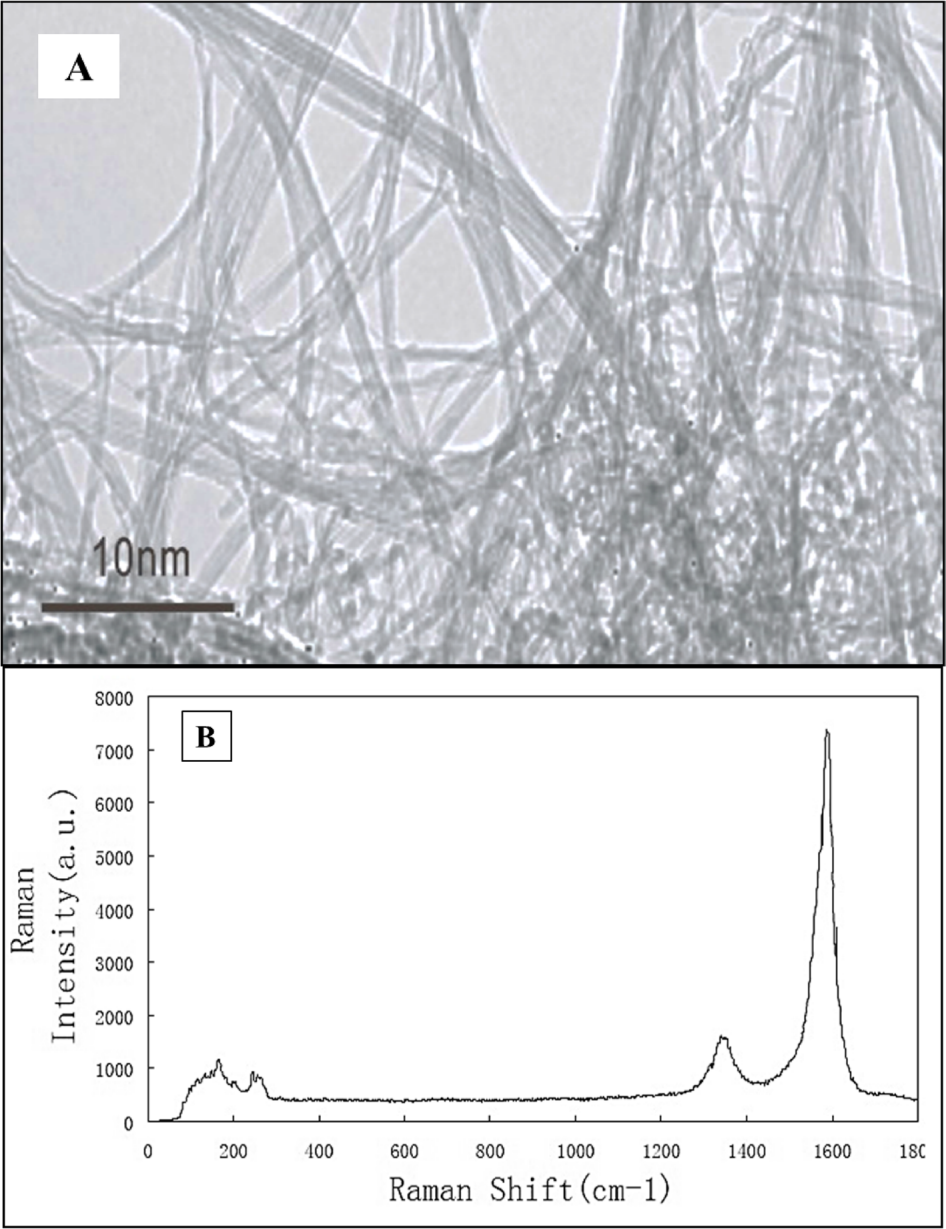
**A** TEM micrograph and (**B**) Raman spectroscopy of SWCNTs used in this study

The SWCNTs (black powder) were described in terms of physical dimensions, elemental components and surface areas (Table 1) by TEM (Fig. 1), Raman spectroscopy (Fig. 1), X ray spectroscopy and diffraction analysis (Aluminum = 0.08%, Sulfur = 0.29%, Cobalt = 2.91%, Chlorine = 0.42%, Carbon = 96.30%).

**Table 1:** Properties of SWCNTs used in the present research

Particle	Purity	Outside diameter (nm)	Inside diameter	Length	Average diameter	Electric Conductivity	Tap density	True density	Thermal Conductivity	I_g_/I_d_
SWCNTs	> 95% wt	1–2 nm	0.8- 1.6 nm	2–30 um	1.1 nm	> 100 S cm^−1^	0.14 g cm-3	~ 2.1 g cm-3	50–200 W/m.K	˃9

### The SWCNTs suspension preparation

Stable aqueous suspensions of SWCNTs were prepared by sonication (40 kHz, 100 W). The suspension was autoclaved for 20 min (120 °C) and was then applied to the growth medium just after preparation.

### Seed sterilization and germination

The seeds of *T. daenensis* were disinfected with NaOCl (2.5% v/v, 10 min) under aseptic conditions. Then, they were washed with sterile deionized water to eliminate NaOCl traces twice. Thereafter, the disinfected seeds were placed in Murashige and Skoog (MS) medium containing agar (0.8%), sucrose (3%) and different doses of SWCNTs (1000, 500, 250, 125 and 62.5 µg ml^−1^). The medium was intended for germination [30] in petri dishes with 10 replicates. Nine seeds were used per each replicate. The vials were maintained in a growth chamber at 25 ± 2 ºC (which had white fluorescent light, 3,000 lx, 16 h photoperiod) and 55–60% relative humidity. The MS medium without SWCNTs was considered as the control.

### Seed germination

The germination of seeds in the growth medium was documented for 25 days. Subsequently, the below formula was used for the germination percentage calculation [31].

$$\mathrm{GP}\hspace{0.17em}=\hspace{0.17em}(\mathrm{germinated\ seeds\ number}/\mathrm{ planted\ seeds\ number})\hspace{0.17em}\times \hspace{0.17em}100$$

### Seedling growth

Responses to SWCNTs by the *T. daenensis* seedlings were studied via morphological, physiological and phytochemical parameters. After germination, *T. daenensis* seedlings were grown in the same conditions for 40 days. This was followed by harvesting the seedlings and recording their morphological properties.

### Enzymes assays

#### Enzyme extracts preparation and assays

Fresh leaves (100 mg) were milled with liquid N_2_ and blended in cold sodium phosphate buffer that contained EDTA (0.1 mM) (1.5 ml, 50 mM, pH 7.8). The mixture was centrifuged for 20 min at 12 000 rpm (4 °C) and the obtained supernatants were used as enzyme extract (PAL, PPO, and POD).

#### Polyphenol oxidase enzyme activity (PPO)

The method of Soliva et al. [32] was used for the calculation of PPO enzyme activity at 410 nm for 3 min. Briefly, the reaction mixture in the volume of 3 ml consist of K-phosphate buffer (0.1 M, 2 ml, pH 7.0), catechol (0.1 M), and enzyme extract (50 µl). The activity of the PPO enzyme was described as unit g^−1^ FW min^−1^.

#### L-Phenylalanine ammonia-lyase enzyme activity (PAL)

Activity of PAL (unit g^−1^ FW) was studied using a method that involved a spectrophotometer at 290 nm [33]. Briefly, the enzyme reaction mixture (1 ml) comprised of L-phenylalanine (0.5 ml, 10 mM), enzyme extract (0.1 ml) and 0.05 M Tris–HCl buffer (pH 8.0). The reactions were stopped by 1 N Hydrogen chloride (0.1 ml).

#### Activity of peroxidase enzyme (POD)

Activity of POD enzyme was determined by monitoring for 1 min at 470 nm (26.6 mM^−1^ cm^−1^) [34]. The reaction mixture comprised of one ml consists of enzyme extract (0.1 ml), phosphate buffer (50 mM, pH 6.8), 15 mM guaiacol and H_2_O_2_ (5 mM).

#### Dehydrogenase enzyme activity (DHA)

Activity of DHA enzyme was measured according red-colored Triphenyl formazan (TPF) formation at 485 nm by a spectrophotometer [35] (Liu et al., 2008). Briefly, young roots were incubated with 2,3,5- triphenyl tetrazolium chloride (TTC) (0.4%) and phosphate buffer (0.06 mol l^−1^, pH 7.0) for 3 h at 37 °C (dark conditions). The reaction of mixtures was stopped by 1 M sulfuric acid.

### Morphological observation by TEM and optical microscopy

*T. daenensis* root cells were studied by TEM and optical microscopy to examine whether SWCNTs could enter the plant cells and cause changes in cell morphology. For optical measurements (Nikon, NI-SS, 934,611, Japan) and TEM imaging (LEO 912AB, Germany), 10-day-old seedlings that had grown on untreated and SWCNTs treated (250 µg ml^−1^) were cut and prefixed in Glutaraldehyde (3.5%). Then, they were rinsed with 0.1 M phosphate buffers (pH 7.0), post-fixed in Osmium Tetroxide (1.0%), dehydrated in increasing alcohol series and mounted in resin. An ultra-microtome equipped with a diamond knife was used for 1000 and 7 nm thick sections preparation. For TEM imaging, copper grids were prepared [36].

### Preparation of methanolic extract

Thyme methanolic extract was prepared by sonication (500 w) of 100 mg in 1000 ml methanol (80%) for 20 min at 40 °C. Then, the mixture was centrifuged for 20 min (13,000 × g, at 25 °C) and its supernatant was used for further analysis (AA, TPC and TFC).

#### Total phenolics (TPC) content

TPC (mg GAE g^−1^ DW) was measured based on Slinkard and Singleton [37] described procedure (the Folin-Ciocalteu method). The results were reported as Gallic acid (GAE, 1000, 500, 250, 125 and 62.5 µg ml^−1^) in a calibration curve (y = 0.0017x—0.1686, *R*^*2*^ = 0.99) at 725 nm. Briefly, methanolic extracts (100 µl) were merged with the Folin-Ciocalteu reagent (1000 µl) (Sigma-Aldrich, Germany) and deionized with distilled water (2000 µl). After 3 min, sodium carbonate (Na_2_CO_3_) (20% W/V) was added to neutralize the mixture.

#### Total flavonoid (TFC) content

TFC was calculated according to AlCl_3_ spectrophotometric assay at 510 nm as previously described by Zhishen et al. [38]. TFC was reported as Quercetin (65.5, 125, 250, 500 and 1000 µg ml^−1^) standard curve (y = 0.0001x + 0.0545, *R*^*2*^ = 0.98) and the results expressed as mg QUE g^−1^ DW. Accordingly, methanolic extract (1 ml) was mixed with 5% NaNO_2_ (300 µl) and deionized water (4 ml). After 5 min, AlCl_3_ (300 µl, 10%), NaOH (2 ml, 1 M) and distilled water (10 ml) were added to the mixture, which was shaken at room temperature (25 °C for 30 min, dark conditions).

### DPPH free radical scavenging activity

Free radical scavenging activities of methanolic samples extracts were analyzed according to Burits and Bucar [39] protocol. Briefly, the reaction mixture was comprised of 2 ml methanol extract and one ml 0.2 mM DPPH methanolic solution. The resultant mix was stored in the dark conditions for one hour (room temperature) and finally read at 517 nm. Synthetic ascorbic acid was used at various concentrations (1000, 750, 500, 400, 300, 200, 100, 50 ppm) to resemble the positive standard. The DPPH inhibition percentage (IP%) was calculated by IP% = (blank absorbance – sample absorbance / blank absorbance) × 100. An extraction solution of methanolic composition (methanol 80%) was used as a control.

### Statistical analysis

A completely randomized design (CRD) with 10 replications was used for current study. The statistical analyses were done using SPSS (IBM Corp) software. The obtained data were described as mean values ± standard deviation (SD). The cluster analysis of un-weighted pair group method was carried out with an arithmetic average (UPGMA), G-plot and correlation analyses (corrplot) using RStudio software [40].

## Results

### Microscopic observations

**Fig. 2:**
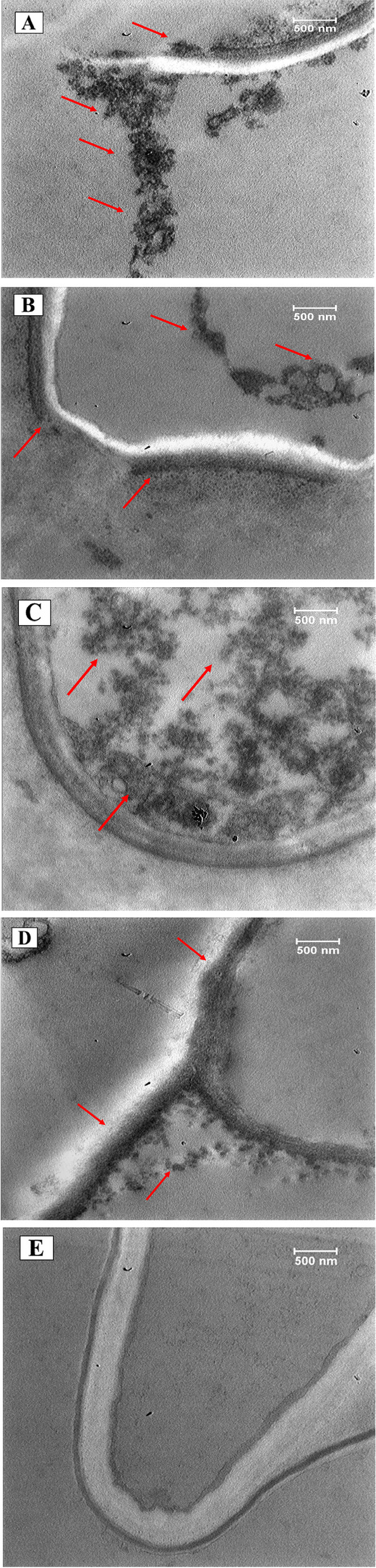
TEM images from *T. daenensis* roots cultivated on MS medium supplemented with SWCNTs (250 µg ml^−1^) (**a**, **b**, **c** and **d**). Images show SWCNTs aggregation (marked with red arrows) in root cells exposed to SWCNTs. **d** TEM images of seedling root grown on medium without SWCNTs

To observe SWCNTs, their uptake and translocation in *T. daenensis* seedlings, optical and TEM microscopy techniques were considered (Fig. 2). TEM and optical imaging can increase our understanding of how SWCNTs affect seedling physicochemical characteristics (Fig. 2). After 10 days of exposure, TEM and optical microscopy analyses were carried out on plants that had been treated with SWCNTs (62.5 µg ml^−1^) and they were compared with untreated control samples (Fig. 2). The aggregation of SWCNTs in filamentous forms is clearly visible in treated cell walls (marked with red arrows), in the cytoplasm and membrane, compared with the control (Fig. 2).

**Fig. 3:**
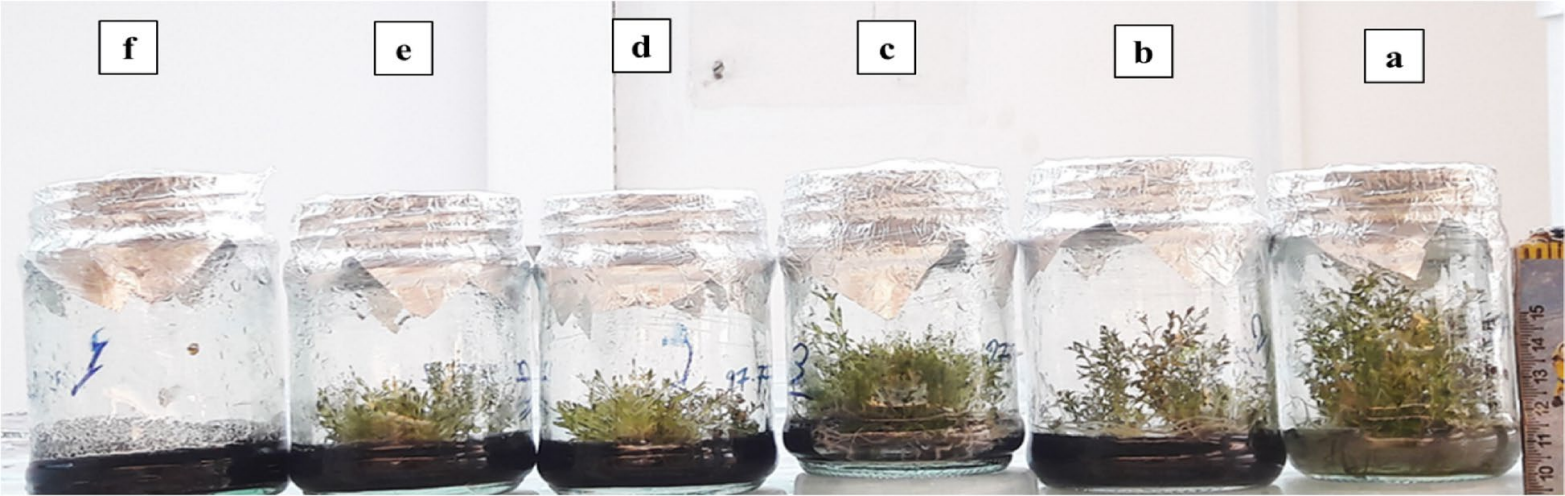
Exposure to SWCNTs in *T. daenensis* seedlings grown in vitro (30-days-old). Exposure to different doses of SWCNTs control (**a**), MS medium treated with 62.5, 125, 250, 500 and 1000 µg ml.^−1^ SWCNTs (**b**, **c**, **d**, **e** and **f**)

**Fig. 4:**
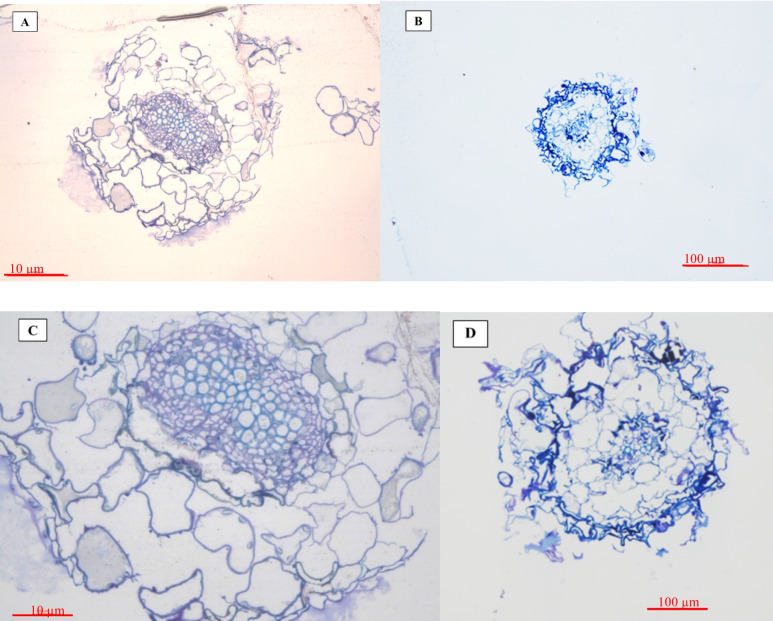
*T. daenensis* roots cross sections in control plants (**A**, **C**) (scale: 10 and 100 µm, respectively) and SWCNTs treated seedlings (250 µg ml^−1^) (**B**, **D**) (scale: 10 and 100 µm respectively). Larger cells and larger root diameters are evident in control seedlings compared to the treated tissues

The ultrastructure of root cells (Figs. 3 and 4) showed apparent changes in response to the SWCNTs treatment. *T. daenensis* seedlings that were not treated with SWCNTs had natural values and features of cell size, cell walls, chloroplast number and chloroplast size. With the addition of SWCNTs to the MS medium, however, cell size shrank and the cell walls were destroyed (Fig. 4). This finding reveals that SWCNTs penetrate *T. daenensis* seedling roots and act like elicitors. They change cells physiologically and increase the accumulation of important pharmaceutical molecules.

### Seed germination percentage, seedling growth and biomass accumulation

Analysis of variance (ANOVA) for morphological traits is outlined in Table 2. Significant differences were obtained following the influence of SWCNTs on germination percentage and seedling development. *T. daenensis* sterile seeds were placed in glasses containing MS medium fortified with SWCNTs at 1000, 500, 250, 125, 62.5 µg ml^−1^ compared to control.

**Table 2:** Germination percentage and biomass variation in *T. daenensis* seedlings treated with SWCNTs

SWCNT (µg ml^−1^)	Germin 2 (%)	Germin 4 (%)	Germin 10 (%)	Germin 25 (%)	Shoot FW (g)	Root FW (g)
0	51.85 ± 5.74 c	61.11 ± 6.09 b	79.86 ± 4.13 b	79.861 ± 4.1 b	0.25 ± 0.16 a	0.25 ± 0.18 a
62.5	86.67 ± 4.97 ab	88.89 ± 9.94 a	88.89 ± 9.07 ab	87.302 ± 9.9 ab	0.15 ± 0.11 b	0.03 ± 0.03 b
125	92.59 ± 9.07 a	88.89 ± 14.05 a	94.44 ± 8.49 a	92.063 ± 10.6 a	0.08 ± 0.05 c	0.02 ± 0.027 b
250	88.89 ± 0.00 ab	77.78 ± 12.17 ab	82.54 ± 8.74 b	80.159 ± 7.7 b	0.05 ± 0.04 c	0.019 ± 0.02 b
500	79.63 ± 14.77 b	64.81 ± 4.54 b	54.17 ± 12.51 c	42.063 ± 13.5 c	0.052 ± 0.05 c	0.03 ± 0.03 b
1000	44.44 ± 11.01 d	53.70 ± 10.92 c	52.78 ± 13.03 c	38.889 ± 9.3 c	0.042 ± 0.05 c	0.02 ± 0.03 b

*Abbreviation: Germin 2, 4, 10 and 25* germination after 2, 4, 10 and 25 days, *shoot FW* shoot fresh weight, *Root FW* root fresh weight

The results indicated a significant increase in germination percentage in response to using 62.5, 125 and 250 µg ml^−1^ in comparison to other treatments and the control group. Furthermore, the germination percentage decreased at SWCNTs higher doses (500 and 1000 µg ml^−1^).

A decrease was observed in the biomass and height of seedlings that grown on MS media with SWCNTs (Tables 2 and 3) in comparison to control samples. It is evident that SWCNTs significantly (p ≤ 0.05) suppressed the fresh and dry biomass accumulation and height of seedlings.

**Table 3:** Root elongation and shoot height variation in *T. daenensis* seedlings treated with SWCNTs

SWCNT (µg ml^−1^)	Root heigth4 (cm)	Shoot height4 (cm)	Shoot length10 (cm)	Shoot length25 (cm)	Shoot length40 (cm)
0	1.5 ± 0.00 a	0.65 ± 0.03 a	1.58 ± 0.1 a	2.2 ± 0.2 a	5 ± 1.4 a
62.5	1.6 ± 0.19 a	0.61 ± 0.1 a	1.5 ± 0.18 a	2.1 ± 0.5 a	3.7 ± 0.5 b
125	1.01 ± 0.09 b	0.51 ± 0.03 b	1.49 ± 0.2 a	2.03 ± 0.5 a	2.70 ± 0.2 c
250	0.59 ± 0.03 c	0.50 ± 0.03 b	1.37 ± 0.42 b	1.8 ± 0.4 b	2.78 ± 0.2 c
500	0.25 ± 0.02 c	0.47 ± 0.06 bc	0.66 ± 0.2 c	1.9 ± 0.3 b	1.8 ± 0.2 d
1000	0.1 ± 0.02 d	0.10 ± 0.018 c	0.31 ± 0.08 d	0.50 ± 0.0 d	0.85 ± 1.0 e

*Abbreviations*: *root heigth4* root height after 4 days, *shoot height 4* shoot height after 4 days, *shoot height 7* shoot height after 7 days, *shoot height 10* shoot height after 10 days, *shoot height 25* shoot height after 25 days, *shoot height 40* shoot height after 40 days

### Phytochemical analyses

Upon SWCNTs application, the amount of TPC significantly increased in the callus, reaching a peak in response to the SWCNTs concentration of 62.5 µg ml^−1^. Concentrations that were lower or higher than 62.5 µg ml^−1^ resulted in lower TPC values. A similar pattern was observed in the case of TFC. The findings revealed that the highest TFC and TPC occurred in response to 62.5 µg ml^−1^ SWCNTs.

Methanolic extracts (80%) were obtained from the callus and their antioxidant activity was calculated by the DPPH reagent. The findings showed that treating the callus samples with 62.5 µg ml^−1^ SWCNTs led to maximum antioxidant content (87.64% ± 0.3) (P ≤ 0.05). Second to this maximum content, the concentration of 125 µg ml^−1^ (80.35% ± 0.8^a^) SWCNTs proved effective (Table 4). Moreover, the results indicated that the antioxidant activity of some treated samples were greater than the effect of 500 µg ml^−1^ ascorbic acid (79.6%) as the positive control. Furthermore, the antioxidant activity of samples positively correlated with the amounts of TFC (r_0.05_ = 0.74) and TPC (r_0.05_ = 0.74) in the callus. These findings imply that TFC and TPC play vital roles in antioxidant activity.

**Table 4:** Supplementation with various concentrations of SWCNTs changed DPPH free radical scavenging activity, total flavonoids (TFC) and total phenolics (TPC) in *T. daenensis* seedlings

SWCNT (µg ml^−1^)	TPC (mg GAE g^−1^ DW)	TFC (mg QUE g^−1^ DW)	DPPH (%)
0	3.08 ± 0.2 a	41.80 ± 1.4 a	75.17 ± 0.9 b
62.5	3.40 ± 0.02 a	42.15 ± 0.9 a	87.64 ± 0.3 a
125	2.72 ± 0.09 b	38.00 ± 2.1 ab	80.35 ± 0.8 a
250	2.87 ± 0.07 b	34.80 ± 0.5 b	74.11 ± 0.7 b
500	1.24 ± 0.03 d	21.95 ± 10.5 c	63.64 ± 0.8c
1000	2.22 ± 0.8 c	13.00 ± 3.0 d	64.82 ± 0.3c
Ascorbic acid (0.5 µg g^−1^)			79.60 ± 0.00

Regarding methanolic extracts and their enzymatic activity, statistically significant differences were recorded among seedlings exposed to SWCNTs in vitro. Significantly, the activity of PAL enzyme proved to have an important role in all samples that were exposed to SWCNTs, in comparison to the control. Moreover, activity of PAL enzyme summit at samples of the control group, reaching 12.51 ± 0.07 mM cm g^−1^ FW. Contradictory, an increase was reported in POD and PPO enzyme activities when higher concentrations of SWCNT were applied (Table 5).

**Table 5:** Activities of PAL, POD, PPO and DHA enzymes in the extracts of *T. daenensis* exposed to different concentrations of SWCNTs

SWCNT (µg ml-1)	PAL (mM cm g^−1^ FW)	POD (µm. cm g^−1^ FW min^−1^)	PPO (mM cm g^−1^ FW min^−1^)	DHA (µm. TPF g^−1^ min^−1^)
0	12.51 ± 0.07 a	12.49 ± 0.10 f	0.50 ± 0.01 c	0.52 ± 0.01 a
62.5	12.05 ± 0.2 a	14.43 ± 0.05 d	0.59 ± 0.00 b	0.47 ± 0.00 a
125	12.44 ± 2.8 a	16.74 ± 0.07 c	0.62 ± 0.01 b	0.18 ± 0.02 b
250	9.81 ± 0.08 b	13.030 ± 0.07 e	0.83 ± 0.08 a	0.08 ± 0.02 c
500	5.46 ± 0.4 c	75.64 ± 0.2 b	0.85 ± 0.01 a	0.07 ± 0.00 c
1000	1.66 ± 0.05 d	90.79 ± 0.06 a	0.81 ± 0.00 a	0.08 ± 0.01 c

*Abbreviations*: *DHA* dehydrogenase, *POD* peroxidase, *PPO* polyphenol oxidase, *PAL* phenylalanine ammonia lyase

**Fig. 5:**
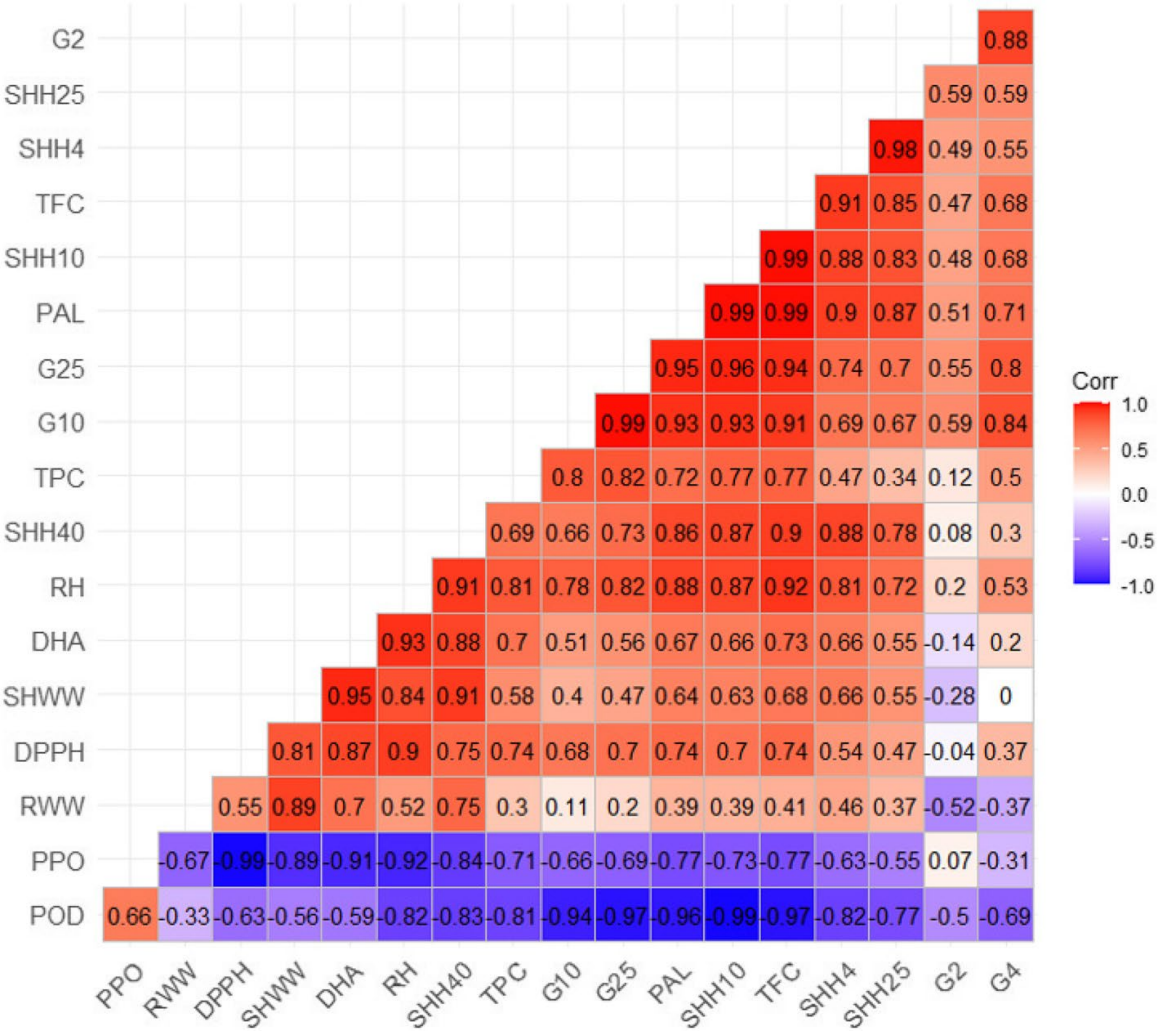
Pearson’s correlation coefficient analysis for seedlings exposed to SWCNTs (G2: germination after 2 days, G4: germination after 4 days, G10: germination after 10 days, G25: germination after 25 days, SHFW: shoot fresh weight, RFW: root fresh weight, RH: root height, SHH 4: shoot height after 4 days, SHH 7: shoot height after 7 days, SHH 10: shoot height after 10 days, SHH: shoot height after 25 days, SHH 40: shoot height after 40 days, PAL: phenylalanine ammonia lyase, TFC: total flavonoids, TPC: total phenolics, PPO: polyphenol oxidase, POD: peroxidase, DHA: dehydrogenase)

DHA enzyme analysis of callus revealed that the decrease in DHA upon application of SWCNTs was concentration-dependent (Table 5). The decrease ultimately leads to less water absorbance and, subsequently, less vegetative biomass. Our analysis revealed that DHA positively correlated with shoot and root dry and fresh weights, PAL (r_0.05_ = 0.67), TFC (r_0.05_ = 0.73), TPC (r_0.05_ = 0.70) and antioxidant activity (r_0.05_ = 0.87) (Fig. 5).

### Cluster analysis

The methods of average linkage and Euclidean distance coefficient analysis were considered for *T. daenensis* seedlings that had been exposed to SWCNTs various doses (Fig. 5). The G-plot analysis showed two main clusters. The first main cluster comprised treatment groups of SWCNTs (62.5, 125 and 250 µg ml^−1^) and control seedlings. Another main cluster comprised treatment groups of 500 and 1000 µg ml^−1^ SWCNT concentrations (Fig. 5).

## Discussion

### Detecting the presence of SWCNTs using microscopy techniques and their effects on cells

TEM imaging was employed to better understand how SWCNTs affect *T. daenensis* seedlings in terms of vegetative and phytochemical properties. For this purpose, seedling root samples of the optimum SWCNTs treatment (62.5 µg ml^−1^) were used. TEM findings revealed that SWCNTs successfully penetrated the cell wall, cytoplasm and membrane of the root tissue. Meanwhile, SWCNTs were not present in images of the untreated samples. Two recent cases of research in the scientific literature indicated that both SWCNTs and MWCNTs entered the callus and seedling root cells of treatment groups, respectively [41]. Relevantly, Martinez-Ballesta et al. [42] reported that MWCNTs penetrated protoplasts of broccoli root cells and led to an overexpression of aquaporins (PIP1s and PIP2s).

Optical microscopic images were taken in addition to TEM imaging to describe xylem and phloem cells. Cross sections of seedling roots (semi thin) were observed in 10 and 50 µm magnifications by a Canon microscope apparatus. The results showed that root diameter, phloem and xylem cell sizes decreased in response to 62.5 µg ml^−1^ SWCNTs exposure. This explains the smaller amount of vegetative biomass in the seedlings of the treatment groups and, in retrospect, greater oxidative damage and stress. Most recently, in a study by Wu et al. [43], the uptake, accumulation and distribution of SWCNTs in crabapple (*Malus hupehensis* Rehd) leaves were detected/confirmed by TEM, and the subsequent impacts of different concentrations of SWCNTs on enzyme activities and genes expression related to plant metabolism were evaluated.

### SWCNTs can change seed germination and morphological properties of seedlings

Nano materials usually affect plants by modifying their growth and development (Lahiani et al. 2015) [44]. Among various nano materials, CNTs have special properties that are characterized by surface chemistry, biocompatibility and attachment affinity. Thus, they have attracted considerable attention among researchers [45]. The same as environmental factors including temperature, light, pH, etc. [46], they can change seed germination and morphological features of seedlings, and several physiological processes (e.g. oxidative stress and cell wall extension) [7].

The current study revealed that SWCNTs can act productively in vitro at low doses (62.5 and 125 µg ml^−1^). They can improve germination percentage in *T. daenensis* seedlings. On the other hand, the negative effects of SWCNTs were observed in seedlings exposed to 500 and 1000 µg ml^−1^ concentrations. In this regard, previous studies indicated that adding high doses of MWCNTs to the MS medium can be toxic for *T. daenensis* and tomato plants, however low concentrations showed positive influence on morphological and physiological procedures [8]. An interesting research by Juárez-Cisneros et al. [47] showed that MWCNTs can be produced after wild fire in forests, thereby improving the seed germination of *Eysenhardtia polystachya*, increasing seedling yield and enhancing morphological properties. Moreover, due to their unique physicochemical characteristics and ability to be readily absorbed by plant cells, CNTs have recently emerged as potential elicitors. This exposure/absorption causes a hormesis (a dose–response phenomenon) effect on plant growth, development and metabolism [48, 49]. In various studies, the inclusion of CNTs in the culture medium has been shown to remarkably improve growth parameters and biomass accumulation in multiple crop species by enhancing the expression of genes involved in root cell elongation (*SLR1* and *RTCS*), water uptake and transport (*NTPIP1*) and cell wall extension (*NtLRX1*) and cell division (*CycB*) [8, 50, 51].

### SWCNTs enhance phytochemicals content and enzymatic features of seedlings

Elicitors (biotic and abiotic) can encourage secondary metabolite accumulation by prompting the overexpression of numerous genes and by affecting materials that are involved in making signal transduction [52]. CNTs reportedly interact with the nitrogen content of plant cell walls via adhesion forces [53], thereby enabling their filamentous structures to attach the plant surface and penetrate the cells [44]. Obviously, CNTs act as elicitors and change gene expression and signal transduction. The proteins and enzymes they encode can alter the pattern of secondary metabolite production in plants. Khodakovskaya et al. [7] studied CNTs and their effects on tomato plants, while reporting that carbon nano materials can act as elicitors and change stress signaling pathways. The current study indicated that SWCNTs can positively affect *T. daenensis* cell when used at low concentrations (62.5 µg ml^−1^), thereby stimulating the production of phytochemical components such as TFC and TPC while enhancing antioxidant activity. This positive correlation in *T. daenensis* seedlings and *Satureja khuzestanica* callus can be seen in agreement with each other in terms of phenolic content, flavonoids, antioxidant activity and PAL enzyme activity [54, 55].

So far, there have been limited accounts of CNTs, their use in agricultural purposes and their impacts on accumulation of secondary metabolite in medicinal plant. A previous study on *T. daenensis* seedlings revealed similar results [41] as in the current study TFC and TPC accumulation occurred in reply to SWCNTs (62.5 µg ml^−1^) to low doses which affected PAL enzyme activity. The decrease in TFC and TPC at higher doses of SWCNTs, however, corresponded with higher POD and PPO activities.

Conversely, SWCNTs are known to induce negative effects on plant cells, thereby resulting in oxidative damage as a consequence of ROS production in a concentration-dependent manner [56]. The present research revealed that SWCNTs can change PPO and POD enzyme activities, while possibly being linked to ROS accumulation in the cells. Furthermore, low concentrations of SWCNTs were observed to increase DHA activity which means an increase in water uptake capability by *T. daenensis*, with more biomass production as a result. The current results are in agreement with a previous research on MWCNTs effects in bean (*Vicia faba* L.) seedlings [57]. The decreased growth observed at higher concentrations of CNTs may be attributed to the potential toxicity of CNTs at elevated doses, which could inhibit in vitro growth [58].

The interaction of CNTs with plant cells stimulates repeated cell divisions and leads to an increase in the production of secondary metabolites [49, 59]. SWCNTs react with plant cell cultures by influencing cell signaling, ROS generation, and secondary metabolism. They initially interact with cell receptors, key-enzymes activity, triggering intracellular processes and stress-related pathways, modulating gene expression, and metabolites biosynthesis [60–62]. These secondary metabolites serve as antioxidant molecules and play vital role in mitigating oxidative/nitrosative stresses and maintaining cellular homeostasis [63–66].

## Conclusions

The present study demonstrates that seed germination can accelerate in response to low doses of SWCNTs (250, 125 and 62.5 µg ml^−1^). Nonetheless, exposure to higher concentrations of SWCNTs in the MS medium showed negative effects on morphological traits of *T. daenensis* seedlings (e.g. shoot height, root elongation, biomass accumulation), and secondary metabolites production compared to the control. This happened because of higher levels of cellular oxidative damage due to increased activity of PPO and POD, which result in higher concentrations of toxic products of oxidation such as quinone. Furthermore, a positive correlation occurred between water availability (DHA activity), seedling height and yield. A significant correlation was also observed among TFC, TPC, radical scavenging capacity and PAL activity. The POD and PPO enzyme activities assisted the plant in coping with an excessive accumulation of free radical molecules. Therefore, this information can increase our knowledge of SWCNTs, to explicate specifically why their effects are important in relation to morpho-physiological and phytochemical processes and how their fate is determined after uptake by the plant system. However, there is still a gap in our knowledge in the field of essential oils production in aromatic medicinal plants upon exposure to SWCNTs, especially at the molecular level, and more research is required in this regard. Also, more research needs regarding nano-carbon uptake, translocation, accumulation, fate and transformation in plant tissues, which will enable effective and safe utilization of carbon nanomaterials for different agricultural applications.

## Data Availability

The raw data of this article will be made available by corresponding author (Prof. Dr. Mansour Ghorbanpour; m-ghorbanpour@araku.ac.ir), according to the personal requests.

## Abbreviations

AA: Antioxidant activity
CNTs: Carbon nanotubes
DHA: Dehydrogenase
DPPH: 2, 2´- Diphenyl-1- picrylhydrazyl
GAE: Gallic acid
MS: Murashige and Skoog
MWCNTs: Multi-walled CNTs
NaOCl: Sodium hypochlorite solution
PAL: L-Phenylalanine Ammonia-Lyase
POD: Peroxidase
PPO: Poly Phenol Oxidase
SD: Standard deviation
SM: Secondary metabolites
SWCNTs: Single walled carbon nanotubes
TEM: Transmission electron microscopy
TFC: Total Flavonoids
TPC: Total Phenolics
TPF: Triphenyl formazan
TTC: 2, 3, 5 Theriphenyl Tetrazolium Chloride
UPGMA: Un-weighted pair group method
